# Strong Associations between Plasma Osteopontin and Several Inflammatory Chemokines, Cytokines, and Growth Factors

**DOI:** 10.3390/biomedicines9080908

**Published:** 2021-07-28

**Authors:** Anders Larsson, Johanna Helmersson-Karlqvist, Lars Lind, Johan Ärnlöv, Tobias Rudholm Feldreich

**Affiliations:** 1 Department of Medical Sciences, Uppsala University, 751 85 Uppsala, Sweden; johanna.helmersson.karlqvist@akademiska.se (J.H.-K.); lars.lind@medsci.uu.se (L.L.); 2 Division of Family Medicine and Primary Care, Department of Neurobiology, Care Sciences and Society (NVS), Karolinska Institutet, 171 77 Stockholm, Sweden; johan.arnlov@ki.se; 3 School of Health and Social Sciences, Dalarna University, 791 88 Falun, Sweden; trf@du.se

**Keywords:** osteopontin, plasma, urine, proinflammatory cytokines, chemokines

## Abstract

Osteopontin is a member of the proinflammatory cytokine network, a complex system that involves many chemokines, cytokines, and growth factors. The aim of the present study was to study the associations between osteopontin and a large number of chemokines, cytokines, and growth factors. We analyzed plasma and urine osteopontin in 652 men from the Uppsala Longitudinal Study of Adult Men (ULSAM) study cohort and compared the levels with the levels of eighty-five chemokines, cytokines, and growth factors. We found significant associations between plasma osteopontin and 37 plasma biomarkers in a model adjusted for age, and 28 of those plasma biomarkers were significant in a model also adjusting for cardiovascular risk factors. There were no significant associations after Bonferroni adjustment between urine osteopontin and any of the studied plasma cytokine biomarkers. This study shows that circulating osteopontin participates in a protein–protein interaction network of chemokines, cytokines, and growth factors. The network contains responses, pathways, and receptor binding interactions relating to cytokines, regulation of the immune system, and also regulation of apoptosis and intracellular signal transduction.

## 1. Introduction

Osteopontin (OPN) was first identified in 1985 [1]. The name, osteopontin, indicates that the protein is expressed in bone, but it is also secreted into plasma and urine and found in several other tissues. Therefore, osteopontin thus functions beyond those related to bone formation. Osteopontin has been implicated in several physiological and pathological processes such as bone turnover [2], cell survival [3], immune regulation and response [4], inflammation [5], ischemia [6], tissue remodeling [7], tumor progression [8], and wound healing [9]. In inflammation, osteopontin acts as a proinflammatory cytokine, modulating the immune response by enhancing expression of Th1 cytokines [10]. Proinflammatory cytokines function within a complex network, stimulating the release of one another, including both cytokine agonists and antagonists. There is limited information on the interactions between osteopontin and other inflammatory chemokines, cytokines, and growth factors. In the present study, we investigated the associations between proinflammatory cytokines reported to be associated with cardiovascular diseases and osteopontin in plasma and urine to increase our knowledge on the interactions between osteopontin and soluble chemokines, cytokines, and growth factors. We used an ELISA from R&D Systems to quantify osteopontin in plasma and urine and correlated the osteopontin levels with the plasma levels of eighty-five chemokines, cytokines, and growth factors.

The aim of this study was to investigate the associations between plasma and urine osteopontin and a broad panel of plasma cytokines using the Proseek Multiplex Cardiovascular I panel. The multiplex proximity extension assays (PEA) simultaneously detected 92 chemokines, cytokines, and growth factors in the same sample.

## 2. Materials and Methods

### 2.1. Patients

The Uppsala Longitudinal Study of Adult Men (ULSAM) study cohort, described in detail at http://www.pubcare.uu.se/ulsam (accessed on 1 June 2021), is an ongoing study since 1970 [11]; the inclusion criteria were 50-year-old male and a resident of Uppsala County, Sweden. The present study uses data from participants who were 77 years old. After exclusion of individuals lacking plasma or urine osteopontin values, 652 participants were included in the plasma part and 457 participants in the urine part of the study. The ULSAM study was approved by the Institutional review board and the Ethics Committee of Uppsala University (Dnr 251/90 (August 1990) and 97/329 (August 1997)).

### 2.2. Clinical Characteristics

Body mass index (BMI) was calculated using standardized methods and expressed in kg/m^2^. Blood pressure was recorded, and data were extracted from a questionnaire completed by the participants regarding socioeconomic status, medical history, smoking habits, medication, and physical activity [8]. The blood pressures were measured at the time as the blood collection. Diabetes mellitus was diagnosed based on fasting plasma glucose (≥7.0 mmol/L) or use of antidiabetic medication.

### 2.3. Osteopontin Measurements

Plasma and urine osteopontin were measured using a commercial sandwich enzyme-linked immunosorbent assay (ELISA) kit (DY1433, R&D Systems, Minneapolis, MN, USA), as previously reported [12]. The limit of quantification (LOQ) of the Osteopontin ELISA was 62 pg/mL. None of the test results were below LOQ. The total coefficient of variation for the ELISA was approximately 6%. The laboratory testing was preformed blinded without knowledge of clinical data.

### 2.4. Proseek Multiplex Measurements

The plasma and urinary chemokines, cytokines, and growth factors were analyzed using Proseek Multiplex Cardiovascular I panel (Olink Bioscience, Uppsala, Sweden). Briefly, 1 µL plasma was mixed with 3 µL incubation mix containing paired antibodies labeled with unique corresponding DNA oligonucleotides. First, the mixture was incubated overnight at 8 °C. Then, 96 µL extension mix containing PEA enzyme and PCR reagents was added, and the samples were incubated for 5 min at room temperature before the plate was transferred to a thermal cycler for 17 cycles of DNA amplification. A 96.96 Dynamic Array IFC (Fluidigm, South San Francisco, CA, USA) was prepared and primed, according to the manufacturer’s instructions. In a separate plate, 2.8 µL of sample mixture was mixed with 7.2 µL detection mix from which 5 µL was loaded into the right side of the primed 96.96 Dynamic Array IFC. The unique primer pairs for each cytokine were loaded into the left side of the 96.96 Dynamic Array IFC, and the protein expression program was run in Fluidigm Biomark reader, according to the instructions for Proseek.

### 2.5. Statistics

Statistical software STATA 15 (StataCorp, College Station, TX, USA) was used in all analyses. Logarithmic transformation was used to promote a normal distribution of osteopontin.

We investigated the associations between plasma and urinary osteopontin, and plasma chemokines, cytokines, and growth factors using the following multivariable linear regression models:

- Age-adjusted model;
- Cardiovascular risk factor model (model A + lipid-lowering treatment, cardiovascular diagnosis, body mass index, diabetes, antihypertensive treatments, systolic and diastolic blood pressure, total and high-density lipoprotein [HDL] cholesterol, and smoking).

In all analyses, urinary and plasma osteopontin were expressed per standard deviation increase. Multiple imputation methods were used to account for the potential influence of missing data with reference to the covariates. Cytokine values above or below the highest and lowest standard points in the Proseek panel were assigned the values of these points. Cytokines with less than 85% of the results in the quantitative range of the Proseek panel were excluded from the comparison. Protein levels in the Proseek panel were measured on a log2 scale and further transformed to a SD scale to be easily comparable. Linear regression analysis was applied to relate plasma and urine osteopontin to the levels of individual cytokines in the Cardiovascular I panel. Analyzing a large number of relationships increases the risk of false positive findings; therefore, the *p*-values were adjusted for multiplicity using the Bonferroni adjustment.

### 2.6. Network Analysis

The protein–protein interaction network for osteopontin and the cytokines significantly associated with osteopontin in the present study were investigated using the online database tool Search Tool for Retrieval of Interacting Genes/Proteins (STRING; https://string-db.org/, accessed on 1 June 2021). The Uniprot numbers were entered in the search engine (multiple proteins) of STRING with the following parameters: organism Homo sapiens, maximum number of interactions was query proteins only, interaction score was set to minimum required interaction score of medium confidence (0.400). In the network figure, each cytokine/chemokine/growth factor is represented by a colored node, and protein–protein interaction and association are represented by an edge visualized as a line. Higher combined confidence scores are represented by thicker lines/edges.

## 3. Results

### 3.1. Study Cohort

The basic characteristics of the study cohort are presented in Table 1. Among the patients, 75 patients had diabetes, three patients with type 1 diabetes and the remaining patients with type 2 diabetes.

### 3.2. Significant Associations between Plasma Osteopontin and Plasma Cytokines

**Figure 1:**
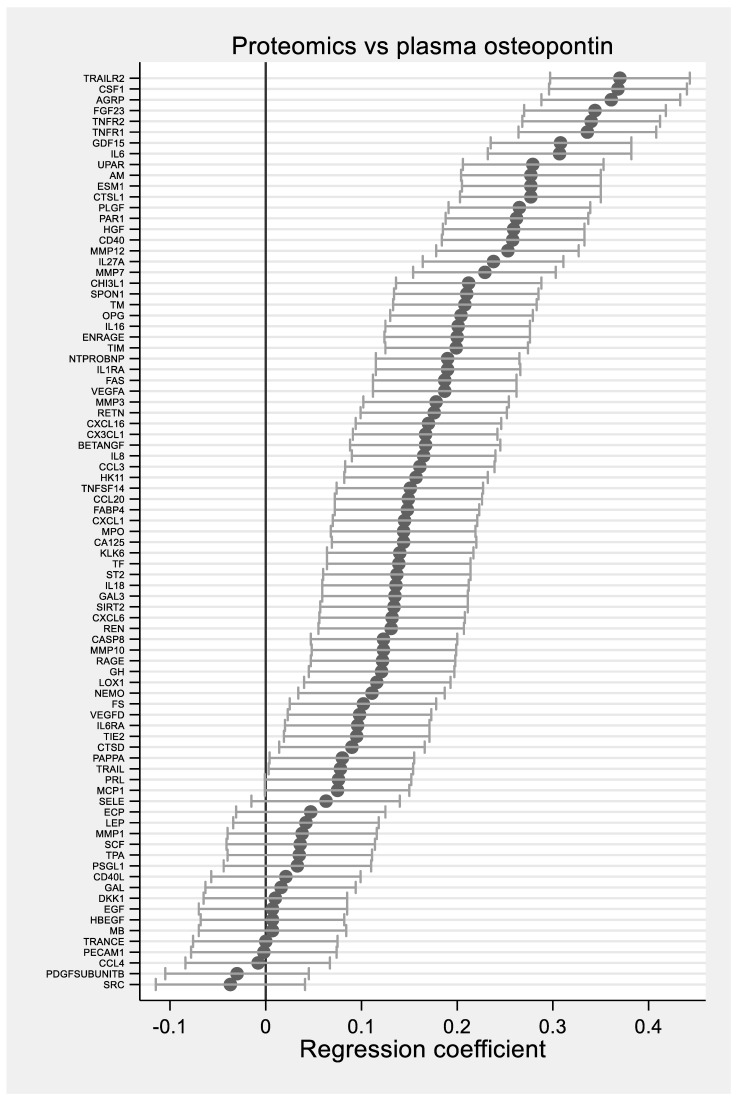
Forest plot showing the associations between plasma osteopontin and 85 biomarkers from the Proseek panel adjusted for age. Data are regression coefficients expressed per SD increase and 95% confidence interval.

There were no osteopontin results that were below LOQ. The multivariate model A showed significant associations between plasma osteopontin and 37 plasma biomarkers in the Proseek panel (Table 2, Figure 1, and Supplementary Table S1). The ten Proseek biomarkers with the strongest correlations to plasma osteopontin were TNF-related apoptosis-inducing ligand receptor 2 (TRAIL-R2) (beta value 0.369), macrophage colony-stimulating factor 1 (0.368), agouti-related protein (0.363), fibroblast growth factor 23 (0.343), tumor necrosis factor receptor 2 (0.340), tumor necrosis factor receptor 1 (0.335), growth differentiation factor 15 (0.308), interleukin 6 (0.307), adrenomedullin (0.278), and endothelial cell-specific molecule 1 (0.275).

The multivariate model B (after adjustment for CVD risk factors) showed that 28 plasma biomarkers remained significantly associated with plasma osteopontin (Table 3 and Supplementary Table S2). The ten Proseek biomarkers with the strongest correlations to plasma osteopontin were macrophage colony-stimulating factor 1 (beta value 0.351), agouti-related protein (0.341), TNF-related apoptosis-inducing ligand receptor 2 (0.339), tumor necrosis factor receptor 1 (0.311), tumor necrosis factor receptor 2 (0.307), fibroblast growth factor 23 (0.315), growth differentiation factor 15 (0.298), interleukin 6 (0.277), endothelial cell-specific molecule 1 (0.262), and adrenomedullin (0.273).

### 3.3. Significant Associations between Urine Osteopontin and Plasma Cytokines

Only tissue-type plasminogen activator, interleukin-1 receptor antagonist protein, thrombomodulin, angiopoietin-1 receptor, kallikrein-6, cathepsin D, and macrophage colony-stimulating factor 1 were significantly associated with osteopontin at *p* < 0.05 in model A (Supplementary Table S3). However, none of the biomarkers remained significantly associated after Bonferroni adjustment. Similarly, in Model B thrombomodulin, macrophage colony-stimulating factor 1, kallikrein-6, and TNF receptor 1 were significantly associated before Bonferroni adjustment but not after the adjustment (Supplementary Table S3).

### 3.4. Network Analysis

The network and enrichment analysis of osteopontin and the 37 proteins significantly associated with osteopontin in Model A based on STRING database identified a protein–protein interaction network that was highly and significantly enriched (protein–protein interaction (PPI) enrichment *p*-value < 1.0 × 10^−16^). Hence, most of these proteins interact with other proteins in the network. Among the 132 terms of biological process (BP) of GO with FDR < 1 × 10^−4^ were responses, pathways, and receptor bindings relating to cytokines, regulation of immune system, as well as regulation of apoptosis and intracellular signal transduction (Supplementary Table S4).

## 4. Discussion

In the present study, we found associations between plasma osteopontin and several inflammatory chemokines, cytokines, and growth factors. In contrast, there were no significant associations found with urine osteopontin after Bonferroni adjustments. We have previously shown that urinary osteopontin was associated with chronic kidney disease while plasma osteopontin was related to cardiovascular disease. Osteopontin has been shown to be highly expressed in the kidney tubule cells (http://www.proteinatlas.org/ENSG00000118785-SPP1/tissue/kidney#imid_7707072, accessed on 1 June 2021). Thus, it is likely that urinary osteopontin mainly reflects a local kidney injury. In contrast, plasma osteopontin is part of the systemic inflammatory response. The analysis indicates that plasma osteopontin is part of a complex protein–protein interaction network according to existing bioinformatic data. The different biological processes that are influenced by the studied network are presented in Supplementary Table S5. The Olink technology is based on the combination of antibodies and DNA amplification. Despite the small sample volumes, the methodology achieves similar or higher sensitivity than traditional ELISAs (Supplementary Table S6).

Osteopontin expression is increased in response to pathophysiological conditions of the heart. Human studies and transgenic mouse models have shown that increased osteopontin production, especially in myocytes, was associated with increased apoptosis and myocardial dysfunction [13]. Experimental studies have indicated that osteopontin played an important role in atherosclerosis development, vascular remodeling, and restenosis [2,14,15,16]. It has also been shown that atherosclerosis modifying therapies with statins or angiotensin II inhibiting drugs reduced circulating osteopontin levels [17,18]. Osteopontin is a key player in the human inflammatory cytokine network with effects on several biological processes. Currently, we have limited knowledge of how osteopontin interacts with other cytokines. To be able to develop disease-specific osteopontin therapies, it is important to know the details of these interactions because we want to modify the disease specific effects without interfering with other biological processes.

The present multiplex protein panel analyzed in plasma was selected to include proteins with known or suggested links to CVD. We have previously found several of these proteins to be inflammatory chemokines, cytokines, and growth factors, which are associated with atherosclerosis and incident CVD [19,20,21]. We have also found plasma osteopontin to be associated with incident CVD [12]. Given the tight coupling between osteopontin and several inflammatory chemokines, cytokines, and growth factors demonstrated both by traditional statistics as well as protein–protein interaction network analysis, it is hard to separate the role of osteopontin in CVD from the other proteins. Future studies using Mendelian randomization are needed to determine which of the associations presented in the present study are causal or not and if osteopontin is causally related to CVD.

A limitation of this study is the fact that the determination of broad cytokine panels is a relatively new concept and therefore, there are no internationally accepted calibrators for most of the studied biomarkers and no well-established reference values. The lack of international calibrators means that each company must develop their own calibrations which makes it difficult to compare results obtained with assays from different manufacturers.

In conclusion, the results of this study show that circulating osteopontin participates in a protein–protein interaction network of chemokines, cytokines, and growth factors. The network contains responses, pathways, and receptor binding interactions relating to cytokines, regulation of the immune system, and also regulation of apoptosis and intracellular signal transduction.

## Figures and Tables

**Table 1:** Basic characteristics of the population (*n* = 652).

Variables	Mean	SD	Min	Max
Age, years	77.6	0.77	75.5	80.7
Body mass index	26.28	3.46	17.6	41.3
Plasma osteopontin, ng/mL	54.6	24.7	10.9	227.4
Urine osteopontin, ng/mL	113.2	64.2	0.727	363.6
Syst blood pressure, mm Hg	150.7	20.4	102	230
Diastolic blood pressure, mm Hg	81.2	9.7	52	115
Total cholesterol, mmol/L	5.40	0.99	2.8	10.2
HDL cholesterol, mmol/L	1.31	0.33	0.37	2.73
	Percentage			
Smoking, %	8.5%			
Diabetes, %	11.5%			
Cardiovascular disease, %	27.9%			
Lipid-lowering treatment, %	17.4%			
Beta-blocker treatment, %	25.8%			
Diuretics treatment, %	16.6%			
Ca channel blocker treatment, %	16.3%			
ACE-inhibitor treatment, %	17.5%			

**Table 2:** Relationship between plasma osteopontin and the 40 cytokines that were most strongly associated with osteopontin. The correlations are adjusted for age. Significant *p*-values after Bonferroni adjustment (*p* = 5.88 × 10^−4^) are highlighted in grey. All proteins were ln-transformed, and then transformed to a SD scale. *n* = 652. Table sorted by *p*-value. ci = confidence interval. The abbreviations are used in Figure 1.

Biomarker	Uniprot	Abbreviation	Beta	Se	Ci Lower	Ci Higher	*p*-Value
Osteopontin	Q3LGB0	SPP1					
Macrophage colony-stimulating factor 1	P09603	CSF1	0.368	0.037	0.295	0.44	6.75 × 10^−22^
TNF-related apoptosis-inducing ligand receptor 2	O14763	TNFRSF10B	0.369	0.038	0.296	0.443	2.50 × 10^−21^
Agouti-related protein	O00253	AGRP	0.363	0.037	0.29	0.436	4.43 × 10^−21^
Tumor necrosis factor receptor 2	P20333	TNFRSF1B	0.34	0.037	0.267	0.413	6.18 × 10^−19^
Tumor necrosis factor receptor 1	P19438	TNFRSF1A	0.335	0.037	0.263	0.407	1.41 × 10^−18^
Fibroblast growth factor 23 (FGF-23)	Q9GZV9	FGF23	0.343	0.038	0.268	0.418	3.02 × 10^−18^
Growth differentiation factor 15	Q99988	GDF15	0.308	0.038	0.234	0.381	1.65 × 10^−15^
Interleukin 6	P05231	IL6	0.307	0.039	0.231	0.383	1.08 × 10^−14^
Adrenomedullin	P35318	ADM	0.278	0.037	0.205	0.351	3.23 × 10^−13^
Endothelial cell-specific molecule 1	Q9NQ30	ESM1	0.275	0.037	0.202	0.348	5.22 × 10^−13^
Urokinase plasminogen activator surface rec	Q03405	PLAUR	0.277	0.038	0.203	0.351	6.82 × 10^−13^
Cathepsin L1	P07711	CTSL	0.274	0.037	0.201	0.348	7.54 × 10^−13^
Placenta growth factor	P49763	PGF	0.267	0.038	0.193	0.341	4.58 × 10^−12^
Proteinase-activated receptor 1	P25116	F2R	0.266	0.038	0.191	0.341	9.75 × 10^−12^
Hepatocyte growth factor	P14210	HGF	0.263	0.038	0.188	0.337	1.14 × 10^−11^
CD 40 ligand	P29965	CD40LG	0.258	0.038	0.183	0.333	2.76 × 10^−11^
Matrix metalloproteinase-12	P39900	MMP12	0.248	0.038	0.173	0.323	1.95 × 10^−10^
Interleukin 27a	Q14213	EBI3	0.236	0.037	0.162	0.309	5.70 × 10^−10^
Matrix metalloproteinase 7	P09237	MMP7	0.233	0.038	0.158	0.308	1.73 × 10^−9^
Thrombomodulin	P07204	THBD	0.211	0.039	0.135	0.286	6.69 × 10^−8^
Chitinase-3-like protein 1	P36222	CHI3L1	0.212	0.039	0.136	0.289	7.10 × 10^−8^
Osteoprotegerin	O00300	TNFRSF11B	0.204	0.038	0.129	0.279	1.44 × 10^−7^
Spondin-1	Q9HCB6	SPON1	0.206	0.039	0.13	0.282	1.61 × 10^−7^
Interleukin 16	Q14005	IL16	0.199	0.038	0.123	0.274	3.29 × 10^−7^
TIM-1/KIM-1	Q96D42	HAVCR1	0.196	0.038	0.121	0.271	3.53 × 10^−7^
Protein S100-A12	P80511	S100A12	0.198	0.039	0.121	0.275	5.74 × 10^−7^
Interleukin-1 receptor antagonist protein	P18510	IL1RN	0.19	0.039	0.113	0.266	1.38 × 10^−6^
Vascular endothelial growth factor A	P15692	VEGFA	0.186	0.038	0.11	0.261	1.68 × 10^−6^
NT-proBNP	P16860	NPPB	0.185	0.038	0.11	0.261	1.79 × 10^−6^
Tumor necrosis factor receptor superfamily member 6	P25445	FAS	0.183	0.039	0.107	0.258	2.54 × 10^−6^
Matrix metalloproteinase 3	P08254	MMP3	0.175	0.039	0.099	0.252	8.53 × 10^−6^
Resistin	Q9HD89	RETN	0.175	0.039	0.098	0.252	9.18 × 10^−6^
Fractalkine	P78423	CX3CL1	0.169	0.039	0.093	0.245	0.0000158
C-X-C motif chemokine 16	Q9H2A7	CXCL16	0.168	0.039	0.092	0.244	0.0000179
Beta-nerve growth factor	P01138	NGF	0.169	0.04	0.091	0.248	0.0000269
Kallikrein-11	Q9UBX7	KLK11	0.159	0.038	0.083	0.234	0.0000412
C-C motif chemokine 3	P10147	CCL3	0.163	0.04	0.085	0.241	0.000051
Interleukin 8	P10145	IL8	0.156	0.039	0.08	0.232	0.0000616
Cancer antigen 125	Q8WXI7	MUC16	0.152	0.039	0.076	0.229	0.0001033
TNFSF14	O43557	TNFSF14	0.152	0.039	0.076	0.229	0.0001105

**Table 3:** Relationship between plasma osteopontin and each of the 40 cytokines that were most strongly associated with osteopontin in model B. Significant *p*-values after Bonferroni adjustment (*p* = 5.88 × 10^−4^) are highlighted in grey. All proteins were ln-transformed, and then transformed to a SD scale. *n* = 652. Table sorted by *p*-value. ci = confidence interval.

Biomarker	Uniprot	Abbreviation	Beta	Se	Ci Lower	Ci Higher	*p*-Value
Osteopontin	Q3LGB0	SPP1					
Macrophage colony-stimulating factor 1	P09603	CSF1	0.351	0.038	0.277	0.424	1.71 × 10^−19^
Agouti-related protein	O00253	AGRP	0.341	0.039	0.264	0.417	2.26 × 10^−17^
TNF-related apoptosis-inducing ligand rec. 2	O14763	TNFRSF10B	0.339	0.04	0.26	0.418	2.23 × 10^−16^
Tumor necrosis factor receptor 1	P19438	TNFRSF1A	0.311	0.039	0.234	0.388	1.08 × 10^−14^
Tumor necrosis factor receptor 2	P20333	TNFRSF1B	0.307	0.039	0.231	0.383	1.13 × 10^−14^
Fibroblast growth factor 23 (FGF-23)	Q9GZV9	FGF23	0.315	0.04	0.236	0.394	1.75 × 10^−14^
Growth differentiation factor 15	Q99988	GDF15	0.298	0.042	0.217	0.38	2.14 × 10^−12^
Interleukin 6	P05231	IL6	0.277	0.039	0.2	0.353	3.47 × 10^−12^
Endothelial cell-specific molecule 1	Q9NQ30	ESM1	0.262	0.038	0.187	0.337	1.67 × 10^−11^
Adrenomedullin	P35318	ADM	0.273	0.04	0.194	0.352	3.36 × 10^−11^
Cathepsin L1	P07711	CTSL	0.242	0.038	0.167	0.316	3.77 × 10^−10^
Urokinase plasminogen activator surface rec	Q03405	PLAUR	0.237	0.039	0.161	0.314	1.92 × 10^−9^
CD 40 ligand	P29965	CD40LG	0.232	0.039	0.156	0.307	2.95 × 10^−9^
Placenta growth factor	P49763	PGF	0.234	0.039	0.157	0.31	4.29 × 10^−9^
Proteinase-activated receptor 1	P25116	F2R	0.228	0.039	0.151	0.305	1.06 × 10^−8^
Hepatocyte growth factor	P14210	HGF	0.226	0.04	0.148	0.305	2.54 × 10^−8^
Interleukin 27a	Q14213	EBI3	0.213	0.038	0.138	0.288	3.51 × 10^−8^
Thrombomodulin	P07204	THBD	0.202	0.038	0.127	0.277	1.73 × 10^−7^
Matrix metalloproteinase 7	P09237	MMP7	0.215	0.041	0.134	0.295	2.49 × 10^−7^
Matrix metalloproteinase-12	P39900	MMP12	0.208	0.041	0.127	0.289	6.64 × 10^−7^
TIM-1/KIM-1	Q96D42	HAVCR1	0.207	0.042	0.125	0.289	8.56 × 10^−7^
Chitinase-3-like protein 1	P36222	CHI3L1	0.193	0.039	0.116	0.27	1.26 × 10^−6^
Osteoprotegerin	O00300	TNFRSF11B	0.186	0.039	0.111	0.262	1.67 × 10^−6^
Interleukin 16	Q14005	IL16	0.183	0.038	0.108	0.258	1.93 × 10^−6^
Spondin-1	Q9HCB6	SPON1	0.173	0.04	0.096	0.251	0.0000148
Tumor necrosis factor receptor superfamily member 6	P25445	FAS	0.167	0.038	0.092	0.242	0.0000154
Protein S100-A12	P80511	S100A12	0.168	0.039	0.091	0.244	0.0000219
Vascular endothelial growth factor A	P15692	VEGFA	0.16	0.038	0.086	0.235	0.0000281
Interleukin-1 receptor antagonist protein	P18510	IL1RN	0.163	0.041	0.083	0.243	0.0000698
Fractalkine	P78423	CX3CL1	0.153	0.039	0.077	0.229	0.0000878
Beta-nerve growth factor	P01138	NGF	0.159	0.04	0.08	0.238	0.0000915
C-X-C motif chemokine 16	Q9H2A7	CXCL16	0.147	0.039	0.07	0.223	0.0001929
NT-proBNP	P16860	NPPB	0.154	0.042	0.073	0.236	0.0002357
Matrix metalloproteinase 3	P08254	MMP3	0.145	0.04	0.066	0.224	0.0003467
Tissue factor	P13726	TF	0.14	0.04	0.062	0.217	0.0004439
Interleukin 8	P10145	IL8	0.135	0.039	0.059	0.211	0.0005121
TNFSF14	O43557	TNFSF14	0.135	0.039	0.059	0.21	0.0005263
Cancer antigen 125	Q8WXI7	MUC16	0.135	0.039	0.058	0.212	0.0006385
C-C motif chemokine 20	P78556	CCL20	0.13	0.039	0.053	0.207	0.0009732
SIR2-like protein 2	Q8IXJ6	SIRT2	0.126	0.039	0.05	0.203	0.0012322

## Data Availability

The datasets used and/or analyzed during the current study are available from the corresponding author on request. This will in most cases also require an ethical permit.

## Supplementary Materials

The following are available online at https://www.mdpi.com/article/10.3390/biomedicines9080908/s1, Table S1: Relationship between plasma osteopontin and the 85 cytokines analyzed in the CVD panel. The correlations are adjusted for age. Table S2: Relationship between plasma osteopontin and the 85 cytokines that were most strongly associated with osteopontin in model B. Table S3: Relationship between urine osteopontin and the 85 cytokines analyzed in the CVD panel. Table S4: Relationship between urinary osteopontin and the 85 cytokines that were most strongly associated with osteopontin in model B. Table S5: The cytokines and the different biological processes that are influenced by the studied networks. Table S6: Lower and upper limits of quantification for the biomarkers included in the Proseek Multiplex Cardiovascular I panel.
